# The influence of intra-alveolar application of honey versus Chlorhexidine rinse on the incidence of Alveolar Osteitis following molar teeth extraction. A randomized clinical parallel trial

**DOI:** 10.4317/jced.55743

**Published:** 2019-10-01

**Authors:** Nedal Abu-Mostafa, Saja Al-Daghamin, Asma Al-Anazi, Nesreen Al-Jumaah, Amenah Alnesafi

**Affiliations:** 1 Assistant Professor in Oral and Maxillofacial Surgery and Diagnostic Science Department, Riyadh Elm University, Kingdom of Saudi Arabia; 2 Dental Interns, Riyadh Elm University, Kingdom of Saudi Arabia

## Abstract

**Background:**

Alveolar Osteitis (AO) is a complication occurs on the post-extraction days that includes pain and disintegrated blood clot. Chlorhexidine (CHX) rinse after extraction is an effective method for decreasing the incidence of AO. Honey has natural antibacterial properties and effectiveness when it is used as a dressing on burns and wounds. However, the effect of intraoral honey dressings on healing is still not adequately studied. This study aimed to compare pain and AO after extraction of a molar tooth in a group of patients who received the intra-alveolar application of Manuka Honey post-operatively with another group who used 0.2% CHX rinse.

**Material and Methods:**

A randomized clinical trial of parallel groups was carried out on 100 patients who had a single molar tooth extraction. They were randomly divided into two groups. Group1 (G1) used 0.2 % CHX twice daily for 7 days. In Group2 (G2), Manuka Honey applied topically by a cotton swab into the socket directly after extraction and on Day3. Re-evaluation, including pain assessment, empty socket, and halitosis was done on day 3 and day 7.

**Results:**

G1 included 43 patients and G2 included 57 patients. Higher grades of pain, more empty sockets, and halitosis were found in G2 than in G1 on day3 and day7 without significant differences. Four cases of AO were found in G1 (9.3%) and 7 cases in G2 (12.3%), without significant difference between the two groups according to Chi-squared tests (*p*=0.753).

**Conclusions:**

The application of Manuka honey in the extraction socket directly after extraction and on day 3 has been found to be insignificantly less efficient in the prevention of AO than CHX rinse twice daily for seven days. However, honey is promising as natural dressing material and further studies are recommended.

** Key words:**Manuka honey, Chlorhexidine, Alveolar Osteitis, extraction, dry socket.

## Introduction

Alveolar Osteitis (AO) or “dry socket” is a complication of tooth extraction that can arise between the first and third day postoperatively. It is characterized by pain at the extraction site and surrounding soft tissue with partially or totally disintegrated blood clot, and it may be associated with halitosis (1). The incidence of AO after dental extractions has been reported in the range of 1 – 30 % (2).

Risk factors for AO include traumatic extraction, tooth fragment remains in the socket (3), and lack of dentist experience (4). Local risk factors are poor oral hygiene and pre-extraction periapical and periodontal infection (5). Other factors relate to the medical history, such as smoking, old age, female gender, oral contraceptives, and weak body defense (1).

Increased local fibrinolysis and disintegration of blood clot in the extraction socket has been suggested as a cause of AO. Accordingly, anti-fibrinolytic agents and tranexamic acid were suggested measures for prevention (2,6). The presence of bacteria has also been considered as an etiological cause of AO. Therefore, antibiotics have been used topically or systematically for prevention (7). Other preventive measures include washing with antiseptic solution like Chlorhexidine (CHX), which has an immediate bactericidal action and a prolonged bacteriostatic action (8). CHX rinse before or after extraction has been reported in the literature as an effective method for decreasing the incidence of AO (9). Furthermore, the intra-alveolar application of bio-adhesive gel of 0.2% CHX showed good results in the prevention of AO (6,10,11,12).

Honey has natural antibacterial properties (3), and it has been used widely as a treatment agent. Recent studies confirmed the effectiveness of honey when used as a dressing on burns and infected or non-infected wounds (13). It helps in granulation and epithelialization as well as shedding of necrotic tissue, and it has an analgesic, antioxidant effect (14). On the other hand, allergy to honey is rare, although allergic reaction might be to the pollen or the bee protein in the honey (15).

This study aimed to compare post-extraction pain, acute alveolar osteitis (AO), and halitosis after extraction of molar teeth in patients who received intra-alveolar application of Manuka Honey with a group of patients who used post-operative 0.2 % chlorhexidine rinse every 12 hours for 7 days.

## Material and Methods

A randomized clinical trial of parallel groups has been carried out on 100 patients who underwent extraction of single molar tooth between October 2016 and August 2017. Extractions were performed by dental interns under close supervision of surgery instructors in the University Clinics.

Inclusion criteria: patients with upper or lower molar teeth indicated for extraction. Exclusion criteria: patients with uncontrolled systemic diseases, epinephrine contraindications, pregnant women, breast-feeding women, and women who were using oral contraceptives. Patients with allergy to CHX, honey, Lidocaine, and Ibuprofen were also excluded. Other exclusion criteria included smoking, presence of acute infection, cystic lesions, traumatic extraction with fractured alveolar bone, extraction requiring bone reduction or root separation, and extractions that lasted more than 30 minutes.

The study followed the CONSORT guidelines and complied with the World Medical Association’s Declaration of Helsinki. The Institutional Review Board approved the study after the complete fulfillment of the scientific and ethical requirements. It was registered in the University Research Center under the registration number FIRP/2016/74. Moreover, the study was registered in the U.S. National Library of Medicine (clinicaltrials.gov), and the ID was (NCT02678104). The objectives of the study were explained to all patients who later signed the informed consents.

The information about the patients, including their name, age, gender, mobile number, file number, and smoking, were collected using the questionnaire. Subsequently, their medical condition, tooth indicated for extraction, pre-operative pain, and halitosis were documented.

-Surgical procedure:

All extractions were done under local anesthesia comprising 2% Lidocaine with 1:80,000 epinephrine. Upper molars were anesthetized using buccal and palatal infiltrations while local anesthesia for lower molars was performed with inferior alveolar nerve block and buccal infiltration. Extractions were done simply by forceps.

Random allocation of the patients into the two parallel groups was done by asking them to choose 1 of the 2 colored cards. The green card indicated Group 1 while the blue indicated Group 2. The patients in Group 1received a bottle of 0.2 % CHX mouthwash and started using it on the second day of extraction, twice daily for 7 days. In Group 2, Manuka Honey was applied topically by a sterilized cotton swab into the extraction socket immediately after tooth extraction. Regular post-operative care and verbal instructions were given to all patients. Additionally, the patients were instructed to take 400 mg of ibuprofen every 8 hours on the 1st and 2nd day of the tooth extraction. No antibiotics were prescribed to the patients in both groups.

-Follow up:

On the third postoperative day (day3), pain assessment was performed by visual analogue scale (VAS). The patients were asked to indicate the pain level from 0 to 10. Score 0 represented no pain while score 10 represented maximum severe pain. Clinical re-evaluation included an assessment of the empty socket with food debris and halitosis. Halitosis was recorded and evaluated by the same investigator in all cases if there was a fetid odor from the patient’s mouth during speech. The intra-alveolar application of honey was repeated in Group 2. On the seventh post-operative day (day7), the same parameters regarding pain, extraction socket, and halitosis were re-evaluated. Alveolar osteitis was diagnosed if the patient presented with pain greater than level 5 associated with empty socket and food debris with or without halitosis on the third day.

Frequencies and percentages were calculated for qualitative data (SPSS software version 22). Chi-square test was applied to compare both groups.

## Results

**Figure 1:**
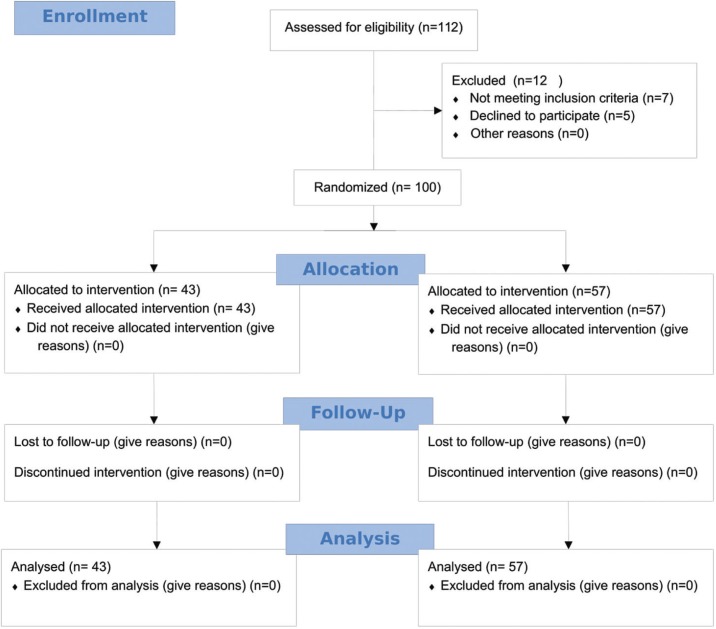
CONSORT flow diagram.

One hundred patients, 48 males (48%) and 52 females (52%), completed the study (Fig. 1). The patients were divided randomly into two groups, with 43 patients in G1 (CHX) and 57 in G2 (Honey). The patients’ age varied from 17 to 69 years, and the mean age was 38.13 years. The mean age of G1 and G2 was 36.9 years and 39.1 years, respectively.

Before extraction, halitosis was noticed in 2 patients in G1 (4.7%) and 3 patients in G2 (5.3%). There was no significant difference between the groups according to Chi-squared tests. On the other hand, the mean of pre-extraction pain in G2 was insignificantly greater than G1 ( Table 1).

**Table 1:**
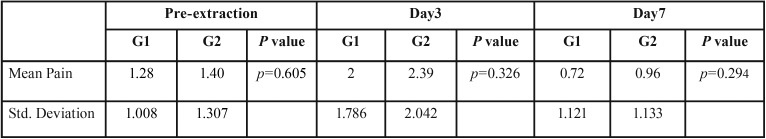
The mean scores of pain before extraction, on day 3, and day 7 according to Independent-Samples T-test.

On day 3, the percentages of empty sockets and halitosis were greater in G2 compared to G1; however, no significant differences were found between the groups according to Chi-squared tests ( Table 1). On day 7, the percentages of empty sockets and halitosis were greater in G2 compared to G1. No significant differences were found between the two groups according to Chi-squared tests ( Table 2).

**Table 2:**
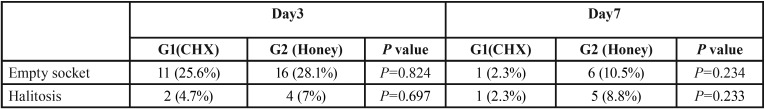
Frequency of Empty socket and Halitosis on day 3 and day 7 according to Chi-squared tests.

On day 3 and day 7, the mean of pain was greater in G2 compared to G1; however, the difference was non-significant (*p*=0.326) and (*p*=0.294), respectively, according to Independent-Samples T-test ( Table 1).

Four AO cases were found in G1 (9.3%) and 7 cases in G2 (12.3%). The difference between the two groups was non-significant (*p*=0.753) according to Chi-squared tests. AO presented in mandible more than in maxilla and more in females compared to males, although without significant differences ( Table 3).

**Table 3:**
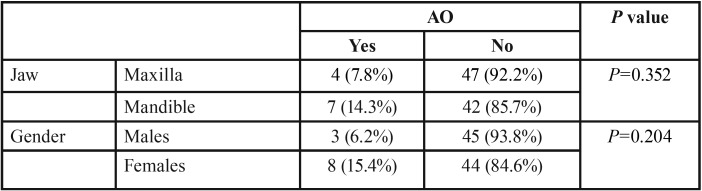
Percentages of AO occurred on the two jaws, and the gender distribution of cases according to Chi-squared tests.

## Discussion

Several studies have thoroughly evaluated honey as dressing material for burn wounds and has been found to be effective in speeding up healing and reducing pain and swelling (13,16,17). However, the effects of intraoral honey dressings on healing and prevention of infection are still unclear. Very few trials in the literature have evaluated honey dressing as a preventive method or treatment agent for AO. In contrast, several studies have evaluated the efficiency of different forms of CHX. It was found that using CHX rinse on the post-surgical extraction days of lower third molar significantly decreases the incidence of AO (4,9,18,19). Additionally, Torres-Lagares *et al.* (6) and Rubio-Palau *et al.* (12) studies have revealed the effectiveness of bio-adhesive 0.2% CHX gel on the reduction of AO when it is used as post-extraction dressing. Other studies have shown greater reduction in the incidence of AO by Intra-alveolar dressing of CHX gel in comparison to CHX rinse (10,11). Chlorhexidine has been the standard agent used for chemical plaque control. However, its adverse reactions include alteration of taste sensation, bad taste, staining of dentures, tongue, gingiva, teeth, and restorations in addition to numbness and stomach upsets (20). Hence, another antiseptic material should be considered.

The study of Elbagoury *et al.* (21) was one of the earliest studies on the effect of honey dressing on socket healing following surgical extraction of impacted third molars. The results showed less pain and fewer occurrences of postoperative complications and swelling in the honey treated group than in the control group.

Singh *et al.* (15) studied the effect of honey as a treatment dressing agent in 54 cases of AO. They found significant reduction in pain, inflammation, hyperemia, edema, and exudation after honey dressing. In the same way, Soni *et al.* (22) found significant decrease in pain, erythema, and swelling after applying honey dressing in 50 cases of AO.

Manuka honey is a monofloral dark honey derived from the manuka tree, Leptospermum scoparium, of the Myrtaceae family that grows as a small tree in New Zealand and eastern Australia (23). Several studies in the literature considered Manuka honey as the gold standard for evaluation of biological and chemical properties of honey (24). In 2019, Al-Khanati NM and Al-Moudallal Y published a split-mouth controlled study on patients who had bilateral impacted lower third molars (25). They performed the surgical extractions of the third molars in two visits. On one side, they applied Manuka honey on the post-extraction socket. Two weeks after, they did the surgical extraction on the other side without putting any medication. The results showed better soft tissue healing and significantly lower pain scores on the honey side than the other side on the 1st and 2nd postoperative days.

We compared 0.2% CHX rinse with Manuka honey as dressing materials on the alveolar sockets after single extraction of upper or lower molars. The investigators applied honey to the extraction socket of the honey group only two times, directly after extraction and on day 3. The results of this study showed higher grades of pain and a greater number of empty sockets in the honey group compared to CHX group on day 3 and day 7, although the differences were non-significant. Similarly, the incidence of AO was greater in the Honey group (12.3%) compare to the CHX group (9.3%), without significant difference between the two groups. Abu-Mostafa *et al.* (11) used the same regimen of post-extraction honey application as we did. The researchers compared the efficiency of post-extraction intra-alveolar application of CHX gel with daily CHX mouth on the incidence of AO. Intra-alveolar application of CHX gel on day 1 and day 3 yielded better results than did daily CHX rinse, although without significant difference. In the present study, the same regimen for honey application has been found to be less effective compared to CHX rinse.

In the literature, female gender was more prone to AO than males (26). In the same way, this study reported more incidence of AO in females (15.4%) than males (6.2%) but without significant difference. On the other hand, higher percentages of AO presented on the mandible which is related to the higher bone density and less blood supply (26).

We found higher percentage of halitosis in honey group compared to CHX group on Day 3 and Day 7, without significant group differences. Similarly, Abu-Mostafa *et al.* (11) found more frequent halitosis with the CHX gel than with CHX rinse. The antibacterial effect of intra-alveolar dressing was limited to the extraction socket, which allowed bacteria to proliferate elsewhere in the oral cavity. In contrast, CHX rinse reduced the microbes count in the oral cavity and kept the mouth moist, decreasing the halitosis. Moreover, the patients used the CHX rinse twice daily; hence, its effect was repeated every 12 hours.

In conclusion, applying honey to the extraction socket only two times, directly after extraction and on the third day, showed insignificant less efficiency in the prevention of AO compared to CHX rinse twice daily for seven days. Because, the differences between both materials in terms of pain, empty sockets, and halitosis were insignificant. Nevertheless, Manuka honey is a promising natural material that needs more trials to identify the most effective regimen of intra-alveolar application and compare it with CHX rinse and intra-alveolar application of CHX gel.
